# Buffalo Medical and Surgical Association

**Published:** 1883-02

**Authors:** Clayton M. Daniels

**Affiliations:** Secretary


### Society Reports.

### BUFFALO MEDICAL AND SURGICAL ASSOCIATION.

*Adjourned Meeting, January 9th, 1883.*

President, Dr. A. R. Davidson, in the Chair. Dr. C. M. Daniels, Secretary.

Present—Drs. Cronyn, Samo, Stockton, Tremaine, Storck, Grove, Brecht, Wyckoff, Coakley, C. A. Ring, Hopkins, Fowler, Hartwig, Kamerling, Mynter, Strong, Bartow, Wall, Howe, Moody, Mackay, Johnson, Mann, and, by invitation, Drs. Elwood, Peterson, Berkes, Frederick and Hubbell.

Applications for membership received from Drs. Edward Clark, E. C. Waldurff, A. A. Hubbell, C. C. Frederick, Geo. E. Fell, H. D. Ingraham and Frederick Peterson, who were duly elected.

Dr. Mynter expected to present two cases with laryngeal affections, but at the last moment the patients positively refused to come to the meeting rooms, and he was, consequently, unable to fulfill his engagement.

Dr. Frederick Peterson exhibited four livers as pathological specimens. One was from a man who had been thrown from a wagon and had, together with fractures of the skull and two ribs, sustained sufficient pressure in his fall upon the abdomen to drive the liver up to the third intercostal space and rupture it. The rupture was of stellar shape, through the capsule, and, as usual in ruptures of the liver, situated near the attachment of the suspensory ligament. About two litres of blood were found in the peritoneal cavity. The man lived three days. The liver was further remarkable in the fatty degeneration which had taken place as a result of the injury. It is rare to see an organ so fatty as this; it is soft, doughy, bloodless and yellow. The man having been apparently a well-nourished, perfectly healthy man, it is, of course, impossible to suppose that the organ was fatty before; and three days is quite sufficient for it to degenerate to such a degree. The speaker had seen the same marked fatty degeneration in liver of a girl of twenty, who died twenty-four hours after eating phosphorus from matches.

The next liver shown was one with ordinary cirrhosis taken from the body of a man accustomed to the use of alcohol in quantities. There had been, of course, the usual enlargement of the spleen, some dropsy of the abdomen, and chronic gastric and intestinal catarrh, from engorgement of the portal circulatory apparatus.

The third liver was one of marked syphilitic hepatitis. In this case were also the sequelæ of portal stasis, as in the case of cirrhosis. The difference between the interstitial hepatitis of alcoholism and that of syphilis is marked by great inflammation of the capsule and numerous peritoneal adhesions, by the large cicatrices and radiating masses of connective-tissue, and by the large, irregular nodules in the syphilitic; while in cirrhosis we have a most even distribution, more uniformity of size of the nodules upon the surface. Further in the interior of this syphilitic liver is to be noticed a large stellate cicatrix whose radii have pulled the liver substance all around up into hard nodules. This is apparently the seat of an old gumma, absorbed and substituted by connective-tissue.

The fourth specimen was one of red atrophy of the liver in heart disease. The man had hypertrophy and dilation of all the heart cavities, and insufficiency of both mitral and tricuspid valves. As a result of tricuspid insufficiency there was venous engorgement and consequent pressure atrophy of the liver. In the liver cells are found particles of reddish pigment, from destruction of red blood corpuscles. The left lobe of the liver is scarcely to be found in this specimen.

Dr. Cronyn presented an instrument which, while not new in itself, was not generally known, and which he thought was of great value in many instances, it being "Parry Durham's improved urethral forceps." Dr. C. had the pleasure of having the *first one* ever made, it being the original obtained from London in 1869. The instrument is exceedingly useful where it is desirable to remove foreign bodies, either from the urethra, uterus or any narrow passage. Dr. C. showed two specimens, one a piece of willow 2½ inches long, which had, by some "unaccountable" way, become firmly imbedded deep in the male urethra, resisting all ordinary means of removal, the other a piece of a knitting needle that had been broken off in the uterus. Both of these foreign bodies were quickly and easily removed with the instrument shown.

Dr. Howe gave the history of a case of *mastoid necrosis* in a child of eight years, it being a sequela of scarlatina. Showed specimens of bone removed.

Dr. Bartow reported a case of "Erysipelas occurring with labor." (The paper is given in the present number of the BUFFALO MEDICAL AND SURGICAL JOURNAL.)

Dr. Hopkins spoke regarding the favorable influence of washing out the uterus, upon high temperature, and said that in his experience thoroughly washing out the bladder had been followed by a lower temperature in a case in which previous irritation of the uterus had failed to procure that result. Referred to a case where a child, sick with scarlet fever, had occupied the same bed with the mother, who passed through an unusually severe labor. No puerperal complications followed.

Also, referring to fears expressed by Dr. Bartow of danger to fœtus where morphia was administered to mother hypodermically, said he had given *two* grains every eight hours for three months before confinement, with no bad results, the maximum quantity being attained two weeks from its first administration.

Dr. Cronyn said that Dr. Bartow's case was full of interest, and to him seemed to prove that there was a unity of poison between scarlatina and the erysipelatous condition, and that pathologists had found the uterus, in similar cases, to be in a like inflammatory condition. Related a case in his own experience.

Dr. Strong regarded the case exceedingly interesting, and thought that the treatment was wise and admirable, especially commending the free use of quinine.

Dr. Bartow replied that he considered that a great portion of benefit obtained was due to the thorough washing out of the uterus.

Dr. Hartwig said that the treatment adopted was doubtless creditable, but in many cases he thought the patient was better protected where no uterine examination was made at all, as proved and advocated by Dr. Alonzo Clark. He wished, also, to take issue with Dr. Cronyn regarding the possibility of the transposition of poisons, believing it impossible for one to propagate other than its own specific disease.

Dr. Cronyn said, as the several fevers had so great a likeness, he should like to have Dr. Hartwig elucidate the difference.

Dr. Hartwig replied that the question was not a difficult one to answer, but it was not timely, at the present late hour, to enter upon its discussion. He had only to remind Dr. Cronyn that the specific poisons of the zymotic diseases always produce their individual and characteristic phenomena, and that all efforts to prove them related have failed.

Some rather active expressions of opinion between Drs. Cronyn and Hartwig followed, reminding some of the older members of the association of the earlier years in its history,

"when discussions waxed warm and were leading features of the meetings."

*Prevailing Diseases*—General pharyngitis, scarlatina, pneumonia, broncho-pneumonia and croup.

The President announced that Dr. Granger would read a paper at the next meeting; subject, "Paresis."

Adjourned.

CLAYTON M. DANIELS, *Secretary.*

